# Associations of Central Sensitization-Related Symptoms with Low Back Pain-Related Factors and Work Status in Caregivers

**DOI:** 10.3390/ijerph191610135

**Published:** 2022-08-16

**Authors:** Hayato Shigetoh, Teppei Abiko, Michie Ohyama, Eiji Sakata, Shin Murata

**Affiliations:** 1 Department of Physical Therapy, Faculty of Health Science, Kyoto Tachibana University, 34 Yamada-cho, Oyake, Yamashina-ku, Kyoto 607-8175, Japan; 2 Non-Profit Organization NPO Fukusiyogunet, Ita, Tagawa 825-0002, Japan

**Keywords:** caregiver, low back pain, psychology, central sensitization-related symptoms, work status

## Abstract

Low back pain (LBP) is associated with psychological factors and central sensitization-related symptoms (CSSs). The relationship between CSSs, LBP-related factors, and work status in caregivers remain unclear. This multicentre, collaborative, cross-sectional study aimed to determine the association between CSS severity, LBP-related factors, and work status in caregivers with LBP. We measured LBP intensity, pain duration, pain sites, CSSs (using the Central Sensitization Inventory-9: CSI-9), psychological factors (using the Pain Catastrophizing and Pain Self-Efficacy scales), and work status (interference, amount of assistance, frequency of assistance, and work environment) in 660 caregivers. CSS severity was categorised as no (CSI-9:0–9), mild (CSI-9:10–19), or moderate/severe (CSI-9:20–36). We further performed multiple comparison analyses and adjusted the residual chi-square to reveal differences between CSS severity groups. Caregivers with more severe CSSs tended to exhibit worse LBP intensity (*p* < 0.01), widespread pain (*p* < 0.01), catastrophic thinking (*p* < 0.01), and pain self-efficacy (*p* < 0.01), and they also tended to experience work interference (*p* < 0.01). Caregivers without CSSs tended to receive a smaller amount of assistance with a lower frequency (*p* < 0.05). The number of participants with an adequate environment and equipment was significantly less in the moderate/severe CSS group (*p* < 0.01). Thus, our findings may suggest that CSS severity is associated with LBP intensity, widespread pain, psychological factors, and work status in caregivers.

## 1. Introduction

Low back pain (LBP) is one of the most common causes of disability worldwide [1]. LBP also interferes with work in many occupations. In particular, caregivers have been reported to have a high prevalence of LBP [2,3]. LBP is an independent factor associated with absenteeism among caregivers [4]. Therefore, LBP prevention programmes for caregivers, such as no-lift policies [5], are being implemented worldwide, and the need for LBP prevention is increasing, particularly in caregivers’ workplaces.

The severity and disability due to LBP are associated with several factors, including psychological, social, and biophysical factors; comorbidities; and pain processing mechanisms [6]. Psychological factors, such as catastrophic thinking and self-efficacy, are often associated with disability in individuals with chronic LBP [7,8,9]. Similarly, a previous study reported that psychological factors are associated with LBP and disability in caregivers [10]. In recent years, some research groups have reported that central sensitization-related symptoms (CSSs) also affect disability in patients with chronic LBP [11,12]. CSSs are an essential factor of pain that have been reported to mediate the relationship between psychological factors and pain [13]. In addition, patients with pain who do not show improvement in CSSs tend not to show improvement in pain [14]. Interestingly, CSSs have been reported as an independent factor associated with a substantially lower rate of work retention [15]. Hence, there is an increasing importance for management to consider CSSs and other psychological factors.

The physical effects of CSSs on caregivers have not been fully clarified, and the association between LBP and CSSs in caregivers is unknown. In addition, the association between caregivers’ work status and CSSs has not yet been clarified. In other words, previous studies have not fully clarified the effects of CSSs on LBP and the work environment among caregivers. Identifying these relationships may lead to reconsideration of LBP management in caregivers. We hypothesised that severe CSSs are associated with negative LBP-related factors and an inadequate work environment. This study aimed to determine the association of CSS severity with LBP-related factors and work status in caregivers with LBP.

## 2. Materials and Methods

### 2.1. Participants and Study Design

**Figure 1:**
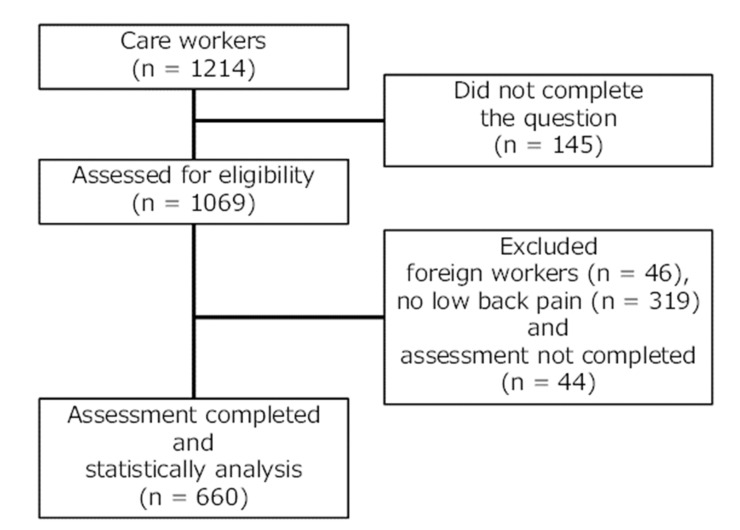
Progression of participants through the assessment including losses to analysis.

This multicentre, collaborative, cross-sectional study was conducted between 1 July and 30 September 2021. A total of 1214 caregivers were recruited from 35 centres. Respondents who self-completed the questionnaires totalled 1064 workers (response rate: 88.1%). The exclusion criteria were foreign workers, no LBP, and an incomplete assessment. The 660 caregivers with LBP who completed the self-administered questionnaire with no missing data were included in the analysis (Figure 1). The sample size of this study was calculated using the G*power analysis program based on an effect size of 0.25, alpha of 5%, and 80% power. According to this calculation, the sample size was determined to be a minimum of 159. Therefore, the sample size of this study is considered adequate. The study protocol conformed to the principles of the Declaration of Helsinki. All participants provided written informed consent before the study commenced. This study was approved by the ethics committee of Kyoto Tachibana University (approval no. 21-39).

### 2.2. Evaluations of the Participants’ Characteristics by Questionnaires

The following characteristics were assessed for each patient: demographics (age, sex, years of employment), question items related to pain, CSSs (using the Central Sensitization Inventory-9 (CSI-9)), cognitive-emotional factors (using the Pain Catastrophizing Scale (PCS) and Pain Self-Efficacy Questionnaire (PSEQ)), and question items related to work status.

The five question items related to pain are as follows: (i) presence/absence of low back pain (yes/no); (ii) duration of LBP (<3/≥3 months); (iii) pain intensity for LBP in the last week (on a numerical rating scale (NRS) (0: no pain, 10: worst pain imaginable)); (iv) pain intensity for LBP before becoming caregivers (by an NRS (0: no pain, 10: worst pain imaginable); and (v) pain sites (head/neck/shoulder/back/hip/knee/ankle) other than the LBP.

The CSI-9, a shorter version of the 25-item CSI, has nine items and was used to assess health-related symptoms of CSSs [16]. Higher scores indicate more severe CSSs.

The PCS was used to assess catastrophic thinking [17]. Higher scores indicate more severe catastrophic thinking.

The PSEQ was used to assess self-efficacy regarding pain [18]. A lower score indicates low self-efficacy.

The four question items related to work status are as follows: (i) interference with work due to LBP (categorised as no interference with work/presenteeism related to LBP/absenteeism related to LBP); (ii) amount of assistance needed for transferring (very little/light assistance/full assistance); (iii) frequency of back-straining movements (movements occurring >10 times per hour or several times in a sequence (often), movements occurring less than or equal to 10 times per hour or continuous movements that are a little burdensome (several), and movements that were less than a few times per day (minimal)); and (iv) environment and equipment to prevent LBP (prevention measures are adequate (adequate), prevention measures are somewhat in place but insufficient (adequate but insufficient), and prevention measures are inadequate (inadequate)).

### 2.3. Statistical Analyses

We divided the participants into three groups based on their CSI-9 score as follows: no, mild, and moderate/severe CSS groups with CSI-9 scores of 0–9, 10–19, and 20–36 points, respectively [16].

We used a Kruskal–Wallis test and multiple comparisons to compare continuous variables between severity groups using Bonferroni’s adjustment method to optimize protection against type 1 errors. The chi-square test was used to compare categorical variables between the severity groups. Then, we calculated the adjusted chi-square residuals considering 1.96 to be a critical value (α = 0.05). Adjusted residuals can be used to determine the significant contributor to a significant chi-square result.

We analysed the following categorical variables: For question items related to LBP, we divided participants into two groups based on LBP duration (acute and chronic LBP with LBP duration < 3 and ≥3 months, respectively). In addition, we divided the participants into three groups based on the LBP intensity (mild, moderate, and severe with an NRS of 1–3, 4–6, and 7–10, respectively). For cognitive-emotional factors, we analysed PCS and PSEQ scores both as categorical and continuous variables. We divided the patients into two groups based on the PCS cut-off score (high and low PCS as PCS score ≥ 30 and <30, respectively) and PSEQ cut-off score (low and high PSEQ as <40 and ≥40, respectively). We divided the participants into two groups based on whether there was interference with work due to LBP (no interference vs. interference (presenteeism and absenteeism)).

Statistical analyses were performed using R (ver. 3.6.1). The level of significance was set at *p* < 0.05.

## 3. Results

### 3.1. Participants’ Characteristics

The participants’ characteristics are summarized in Table 1. The proportion of participants according to CSS severity was 21.8% (no CSSs), 57.6% (mild CSSs), and 20.6% (moderate/severe CSSs). The adjusted residuals for each CSS group are listed in Table 2. Age and years of employment did not significantly differ between the CSS severity groups. In the residual analysis by sex, males were substantially higher, and females were significantly lower in the no CSS group.

### 3.2. Comparison of Pain-Related Factors According to CSS Severity Groups

**Figure 2:**
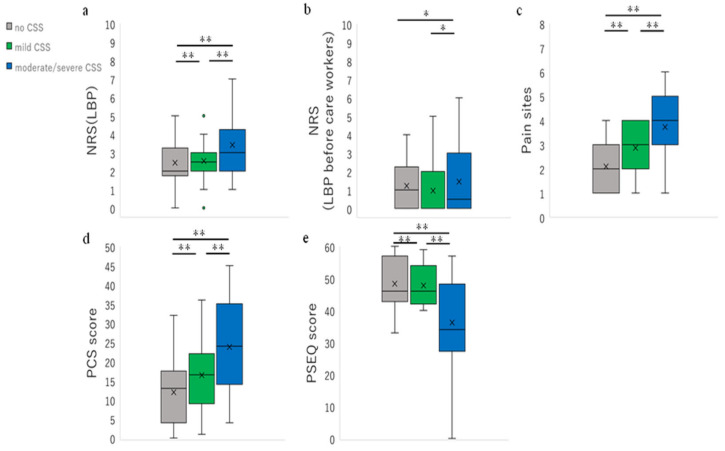
The difference in pain-related factors between CSS severity groups. (**a**) The difference in NRS (LBP) between CSS severity groups. (**b**) The difference in NRS (LBP before becoming caregivers) between CSS severity groups. (**c**) The difference in pain sites between CSS severity groups. (**d**) The difference in PCS scores between CSS severity groups. (**e**) The difference in PSEQ scores between CSS severity groups. **: *p* < 0.01, *: *p* < 0.05.

In terms of pain duration, the number of caregivers with acute LBP was significantly more than those with chronic LBP in the no CSS group (Figure 2), but was significantly lower in the moderate/severe CSS group (Table 2). LBP intensity was significantly higher in the moderate/severe CSS group compared to the no CSS and mild CSS groups (Figure 2). We found similar results for LBP severity and LBP intensity before becoming caregivers. The cognitive-emotional factors (PCS and PSEQ) were significantly worse in the moderate/severe CSS group than in the no and mild CSS groups. The mild CSS group also showed significantly worse cognitive-emotional factors than the no CSS group (Figure 2). Moreover, the cut-off for the participants was significantly higher in the moderate/severe CSS group in the residual analysis (Table 2).

### 3.3. Comparison of Work Status between CSS Severity Groups

Regarding interference with work due to LBP, the number of participants who experienced interference with work was significantly higher in the moderate/severe CSS group. Regarding the amount of assistance needed for transferring, the number of participants requiring very little assistance was significantly higher in the no CSS group. The frequency of back-straining movements was significantly more in the moderate/severe CSS group. The number of participants with an adequate environment and equipment was significantly less in the moderate/severe CSS group.

## 4. Discussion

We used multiple comparison analyses and adjusted chi-square residuals to investigate the relationships between CSSs, LBP-related factors, and work status in caregivers. The results demonstrated that caregivers with severe CSSs tended to have worse LBP intensity, widespread pain, catastrophic thinking, and pain self-efficacy. Regarding work status, caregivers with more severe CSSs tended to experience interference with work. In addition, caregivers with no CSSs tended to receive a smaller amount of assistance and assistance frequency. Interestingly, the number of participants with an adequate environment and equipment was significantly less in the moderate/severe CSS group.

This study aimed to investigate the association between CSS severity and LBP-related factors. In previous studies, severe CSSs were associated with pain severity and chronicity [19,20]. It was also reported that CSSs are associated with a widespread pain area [19]. Recently, nociplastic pain was defined as a pathological classification of pain [21], and the duration of pain, area of pain, and comorbidities corresponding to CSSs were used as indices in the diagnosis algorithm [22]. In the present study, characteristics that were similar to nociplastic pain were shown, especially for moderate/severe CSS caregivers. In addition, it was suggested that psychological factors, such as self-efficacy and catastrophizing, are associated with LBP severity through cognitive-emotional sensitization [23]. Focusing on biomechanical factors, the job of caregivers is similar to other jobs that involve heavy lifting tasks and prolonged working in an uncomfortable posture [24]. However, as the job of caregivers targets people, cognitive-emotional and interpersonal sensitization may be more affected by pain [23]. An association between LBP intensity and psychological factors, focusing on the severity of CSSs in caregivers with LBP, has not been reported. In this study, CSS severity was a factor affecting LBP severity, widespread pain, and psychological factors, suggesting the importance of pain management that considers CSSs.

Another feature of this study was the investigation of the relationship between CSS severity and work status among caregivers. The results of the present study showed that many caregivers with moderate/severe CSSs reported interference with work. CSSs have been considered a factor associated with the difficulty of work retention in a previous study [15], and the result is similar to that reported for the caregivers in this study. Interestingly, the study also found an association between CSS severity and the burden of assistance needed for transferring, as well as an association with the work environment. Contemporary theories of movement and pain-related factors propose a model in which a physical load is associated with the sensitization of the nervous system [25]. Hence, the present study suggests that work environment management for caregivers may help alleviate CSSs and improve interference with work.

The present study is the first to demonstrate an association between CSSs, LBP-related factors, and work status in caregivers with LBP. Previous studies have not fully clarified the effects of CSSs on LBP and the work environment among caregivers. The present study is unique because it comprehensively examined the effects on LBP and work status, including CSSs. Our findings may suggest that CSS severity is associated with LBP severity, psychological factors, and work status, including interference with work. Focusing on CSS management, the effectiveness of pain neuroscience education and lifestyle supervision has been reported [26,27,28]. Considering the points of practical implication in this study may help to develop and manage the work status of caregivers as a follow up to CSS symptoms. In recent years, approaches to reducing the burden on caregivers by educating them on appropriate caregiving methods using welfare equipment have become widespread, and their effectiveness in preventing LBP from worsening has been reported [29]. Along with lifestyle guidance and patient education that takes CSSs into account, management of the work environment and equipment for caregivers, such as the no-lifting care policy, may help improve interference with the work of caregivers.

This study has several limitations. (1) The outcomes were assessed using self-reported questionnaires, which might have resulted in subjective bias. Especially for the survey on the work environment and equipment, it is possible that individual criterion bias affected the verbal rating scale because of the lack of specific criteria (types and number of welfare equipment, etc.). (2) The current study used a cross-sectional study design, and it was not possible to design an intervention based on a longitudinal course. Examining causal relationships based on a longitudinal course is necessary in future studies. Revealing this causal relationship may lead to the development of better management for individuals and institutions, including CSS support.

## 5. Conclusions

To our knowledge, this is the first study to investigate associations between CSSs, LBP-related factors, and work status in caregivers with LBP. Our findings may suggest that CSS severity is associated with LBP intensity, widespread pain, psychological factors, and work status, including interference with work; this knowledge can help improve the management of caregivers with LBP.

## Figures and Tables

**Table 1:** Characteristics according to the CSS severity group.

Variables	No CSSs (*n* = 144, 21.8%)	Mild CSSs (*n* = 380, 57.6%)	Moderate/Severe CSSs (*n* = 136, 20.6%)	*p* Value
Age (years)	44.0 (21.0)	43.0 (18.0)	44.0 (16.5)	N.S.
Sex (male: female)	57:87 (39.6%:60.4%)	117:263 (30.8%:69.2%)	41:95 (30.1%:69.9%)	N.S.
Years of employment	10.0 (11.0)	11.0 (11.0)	11.0 (12.0)	N.S.
Pain duration (acute/chronic LBP (%))	79/65 (54.9%:45.1%)	132/248 (34.7%:65.3%)	29/107 (21.3%:78.7%)	*p* < 0.001
Number of pain sites (including LBP)	2.0 (2.0)	3.0 (2.0)	4.0 (2.0)	*p* < 0.001
LBP intensity (NRS: 0–10)	3.0 (3.0)	4.0 (2.0)	5.0 (3.3)	*p* < 0.001
Severity of LBP: mild/moderate/severe (%)	94/42/8 (65.2%/29.2%/5.6%)	155/162/63 (40.8%/42.6%/16.6%)	34/65/37 (25.0%/47.8%/27.2%)	*p* < 0.001
LBP intensity before becoming caregivers (NRS: 0–10)	1.0 (2.0)	1.0 (2.0)	1.0 (3.0)	*p* < 0.05
CSI-9 (0–36)	7.0 (3.0)	15.0 (5.0)	23.0 (4.0)	*p* < 0.001
PCS (0–52)	15.0 (17.0)	20.0 (13.3)	29.5 (14.0)	*p* < 0.001
PCS ≥ 30 (%)	13 (9%)	68 (17.9%)	68 (50%)	*p* < 0.001
PSEQ (0–60)	43.5 (21.3)	37.0 (15.0)	30.5 (19.0)	*p* < 0.001
PSEQ ≤ 40 (%)	61 (42.4%)	229 (60.3%)	96 (70.6%)	*p* < 0.001
Interference with work due to LBP (no interference vs. interference with work)	85/59 (59.0%/41.0%)	168/155/57 (44.2%/40.8%/15.0%)	51/64/21 (37.5%/47.1%/15.4%)	*p* < 0.001
Amount of assistance needed for transferring (very little/light assistance/full assistance) (%)	13/36/95v (9%/25%/66%)	16/364 (4.2%/95.8%)	3/133 (2.2%/97.8%)	*p* < 0.05
Frequency of back-straining movements (often/several/minimal) (%)	52/58/34 (36.1%/40.3%/23.6%)	105/178/97 (105%/178%/97%)	24/61/51 (24%/61%/51%)	*p* < 0.01
Environment and equipment to prevent LBP (adequate/adequate but insufficient/inadequate) (%)	32/90/22 (22.2%/62.5%/15.3%)	65/235/80 (17.1%/61.8%/21.1%)	15/86/35 (11.0%/63.3%/25.7%)	N.S.

Data are reported as N (%) for categorical variables and median (interquartile range) for continuous variables. % indicates the percentage according to the CSS severity group. The *p* values are based on the Kruskal–Wallis test and chi-square test. CSSs, central sensitization-related symptoms; LBP, low back pain; PCS, Pain Catastrophizing Scale; PSEQ, Pain Self-Efficacy Questionnaire; N.S., not significant; IQR, interquartile range.

**Table 2:** Adjusted chi-square residuals for significant difference in variables among CSS severity groups.

Variables	No CSSs (*n* = 144)	Mild CSSs (*n* = 380)	Moderate/Severe CSSs (*n* = 136)
Sex (male/female)	2.03a/−2.03a	−1.14/1.14	−0.68/0.68
Acute/chronic LBP	5.22a/−5.22a	−1.01/1.01	−4.09a/4.09a
Severity of LBP (mild/moderate/severe)	6.14a/−3.20a/−3.96a	−1.26/1.14/0.17	−4.73a/1.87/3.84a
PCS score (<30/≥30)	4.40a/−4.40a	3.35a/−3.35a	−8.59a/8.59a
PSEQ score (≥40/<40)	−4.40a/4.40a	1.08/−1.08	3.21a/−3.21a
Interference with work due to LBP (no interference/interference with work)	3.53a/−3.53a	−1.11/1.11	−2.25a/2.25a
Amount of assistance needed for transferring (very little/light assistance/full assistance) (%)	2.64a/−0.24/−1.01	−0.89/1.10/−0.63	−1.61/−1.11/1.80
Frequency of back-straining movements (often/several/minimal) (%)	2.64a/−1.29/−1.20	0.14/1.11/−1.37	−2.87a/−0.04/2.91a
Environment and equipment to prevent LBP (adequate/adequate but insufficient/inadequate) (%)	1.90/0.06/−1.83	0.11/−0.27/0.22	−2.07a/0.26/1.60

a indicates a significant difference between groups. CSSs, central sensitization-related symptoms; LBP, low back pain; PCS, Pain Catastrophizing Scale; PSEQ, Pain Self-Efficacy Questionnaire.

## Data Availability

The data presented in this study are available on request from the corresponding author. The data are not publicly available due to privacy and ethical restrictions.

## References

[1] Wu A., March L., Zheng X., Huang J., Wang X., Zhao J., Blyth F.M., Smith E., Buchbinder R., Hoy D. (2020). Global low back pain prevalence and years lived with disability from 1990 to 2017: Estimates from the Global Burden of Disease Study 2017. Ann. Transl. Med..

[2] Alexopoulos E.C., Burdorf A., Kalokerinou A. (2006). A comparative analysis on musculoskeletal disorders between Greek and Dutch nursing personnel. Int. Arch. Occup. Environ. Health.

[3] Andersen L.L., Clausen T., Mortensen O.S., Burr H., Holtermann A. (2012). A prospective cohort study on musculoskeletal risk factors for long-term sickness absence among health care workers in eldercare. Int. Arch. Occup. Environ. Health.

[4] Faber A., Giver H., Strøyer J., Hannerz H. (2010). Are low back pain and low physical capacity risk indicators for dropout among recently qualified eldercaregivers? A follow-up study. Scand. J. Public Health.

[5] Engkvist I.L. (2006). Evaluation of an intervention comprising a No Lifting Policy in Australian hospitals. Appl. Ergon..

[6] Hartvigsen J., Hancock M.J., Kongsted A., Louw Q., Ferreira M.L., Genevay S., Hoy D., Karppinen J., Pransky G., Sieper J. (2018). What low back pain is and why we need to pay attention. Lancet.

[7] Ferrari S., Chiarotto A., Pellizzer M., Vanti C., Monticone M. (2016). Pain Self-Efficacy and Fear of Movement are Similarly Associated with Pain Intensity and Disability in Italian Patients with Chronic Low Back Pain. Pain Pract..

[8] Marshall P.W.M., Schabrun S., Knox M.F. (2017). Physical activity and the mediating effect of fear, depression, anxiety, and catastrophizing on pain related disability in people with chronic low back pain. PLoS ONE.

[9] Wertli M.M., Eugster R., Held U., Steurer J., Kofmehl R., Weiser S. (2014). Catastrophizing-a prognostic factor for outcome in patients with low back pain: A systematic review. Spine J..

[10] Yoshimoto T., Oka H., Fujii T., Kawamata K., Kokaze A., Koyama Y., Matsudaira K. (2019). Survey on chronic disabling low back pain among caregivers at nursing care facilities: A multicenter collaborative cross-sectional study. J. Pain Res..

[11] Kosińska B., Tarnacka B., Turczyn P., Gromadzka G., Malec-Milewska M., Janikowska-Hołowenko D., Neblett R. (2021). Psychometric validation of the Polish version of the Central Sensitization Inventory in subjects with chronic spinal pain. BMC Neurol..

[12] Neblett R., Hartzell M.M., Williams M., Bevers K.R., Mayer T.G., Gatchel R.J. (2017). Use of the Central Sensitization Inventory (CSI) as a treatment outcome measure for patients with chronic spinal pain disorder in a functional restoration program. Spine J..

[13] Shigetoh H., Tanaka Y., Koga M., Osumi M., Morioka S. (2019). The Mediating Effect of Central Sensitization on the Relation between Pain Intensity and Psychological Factors: A Cross-Sectional Study with Mediation Analysis. Pain Res. Manag..

[14] Shigetoh H., Koga M., Tanaka Y., Morioka S. (2020). Central sensitivity is associated with poor recovery of pain: Prediction, cluster, and decision tree analyses. Pain Res. Manag..

[15] Choi Y. (2014). An Examination of the Validity of the Central Sensitization Inventory with Chronic Disabling Occupational Musculoskeletal Disorders.

[16] Nishigami T., Tanaka K., Mibu A., Manfuku M., Yono S., Tanabe A. (2018). Development and psychometric properties of short form of central sensitization inventory in participants with musculoskeletal pain: A cross-sectional study. PLoS ONE.

[17] Sullivan M.J., Bishop S.R., Pivik J. (1995). The pain catastrophizing scale: Development and validation. Psychol. Assess..

[18] Adachi T., Nakae A., Maruo T., Shi K., Shibata M., Maeda L., Saitoh Y., Sasaki J. (2014). Validation of the Japanese version of the pain self-efficacy questionnaire in Japanese patients with chronic pain. Pain Med..

[19] van der Noord R., Paap D., van Wilgen C.P. (2018). Convergent validity and clinically relevant categories for the Dutch Central Sensitization Inventory in patients with chronic pain. J. Appl. Biobehavioral. Res..

[20] van Wilgen C.P., Vuijk P.J., Kregel J., Voogt L., Meeus M., Descheemaeker F., Keizer D., Nijs J. (2018). Psychological Distress and Widespread Pain Contribute to the Variance of the Central Sensitization Inventory: A Cross-Sectional Study in Patients with Chronic Pain. Pain Pract..

[21] Kosek E., Cohen M., Baron R., Gebhart G.F., Mico J.A., Rice A.S.C., Rief W., Sluka A.K. (2016). Do we need a third mechanistic descriptor for chronic pain states?. Pain.

[22] Kosek E., Clauw D., Nijs J., Baron R., Gilron I., Harris R.E., Mico J.A., Rice A.S.C., Sterling M. (2021). Chronic nociplastic pain affecting the musculoskeletal system: Clinical criteria and grading system. Pain.

[23] English B. (2014). Neural and Psychosocial Mechanisms of Pain Sensitivity in Fibromyalgia. Pain Manag. Nurs..

[24] Tan S., Muniandy Y., Vasanthi R. (2021). Prevalence of musculoskeletal disorders and associated work-related risk factors among pastry chefs in malacca, malaysia. Int. J. Aging Health Mov..

[25] Hodges P.W., Smeets R.J. (2015). Interaction between pain, movement, and physical activity: Short-term benefits, long-term consequences, and targets for treatment. Clin. J. Pain.

[26] Galan-Martin M.A., Montero-Cuadrado F., Lluch-Girbes E., Coca-López M.C., Mayo-Iscar A., Cuesta-Vargas A. (2020). Pain neuroscience education and physical therapeutic exercise for patients with chronic spinal pain in spanish physiotherapy primary care: A pragmatic randomized controlled trial. J. Clin. Med..

[27] Malfliet A., Kregel J., Coppieters I., De Pauw R., Meeus M., Roussel N., Cagnie B., Danneels L., Nijs J. (2018). Effect of pain neuroscience education combined with cognition-targeted motor control training on chronic spinal pain a randomized clinical trial. JAMA Neurol..

[28] Nijs J., Leysen L., Vanlauwe J., Logghe T., Ickmans K., Polli A., Malfliet A., Coppieters I., Huysmans E. (2019). Treatment of central sensitization in patients with chronic pain: Time for change?. Expert Opin. Pharmacother..

[29] Iwakiri K., Sotoyama M., Takahashi M., Liu X., Koda S., Ichikawa K. (2018). Effectiveness of re-education based on appropriate care methods using welfare equipment on the prevention of low back pain among care workers: A 1.5 year follow-up study. Ind. Health.

